# Negotiating social medicine in a postcolonial context: Halfdan Mahler in India 1951–61

**DOI:** 10.1017/mdh.2023.11

**Published:** 2023-01

**Authors:** Niels Brimnes

**Affiliations:** Department of History and Classical Studies, Aarhus University, Jens Chr. Skou’s Vej 5, DK-8000 Aarhus C, Denmark.

**Keywords:** Halfdan Mahler, social medicine, India, primary health care, Bhore Committee, World Health Organization

## Abstract

This article investigates how World Health Organisation (WHO) Director-General Halfdan Mahler’s views on health care were formed by his experience in India between 1951 and 1961. Mahler spent a large part of the 1950s in India assigned as WHO medical officer to tuberculosis control projects. It argues that Mahler took inspiration from the official endorsement of the doctrine of social medicine that prevailed in India; even if it was challenged by an increasing preference for vertical, techno-centric campaigns. It shows how, from the outset, Mahler was remarkably hostile towards the highly skilled, clinically oriented doctors, but embraced prevalent ideas of community participation. It suggests that Mahler – although he remained silent on the issue – was impressed by the importance and resilience of indigenous traditions of medicine, despite hostility from leading political figures. In this way, the article attempts to establish links to Mahler’s advocacy of primary health care in the 1970s. A broad approach to health, scepticism toward clinically oriented doctors, preference for simple technologies and community participation, as well as an accommodating attitude towards indigenous practitioners, were all features of primary health care, which correlate well with views developed by Mahler as he negotiated social medicine in India between 1951 and 1961.

In 1951, Danish doctor Halfdan Mahler arrived in New Delhi. He was 28 years old and had been assigned on a World Health Organisation (WHO) mission to supervise India’s mass BCG vaccination campaign against tuberculosis. Mahler left the campaign in the summer of 1955 but returned to India in early 1959 on another WHO secondment. This time he was sent to the southern city of Bangalore to supervise the establishment of the National Tuberculosis Institute (NTI) and to contribute to the design of India’s National Tuberculosis Programme (NTP), which during the 1960s came to be seen as a ‘model programme’ in tuberculosis control to be followed by other developing countries. After two years in Bangalore, Mahler, in 1961, joined the tuberculosis section at the WHO headquarters in Geneva and became chief of the section a year later. Mahler continued to have a significant career within the WHO and served as its Director-General between 1973 and 1988.^2^

Mahler’s tenure as Director-General is closely associated with the vision ‘Health for all by the year 2000’ and the primary health care strategy, which the WHO and UNICEF developed during the 1970s. Primary health care favoured a holistic and inter-sectorial approach to health, promoted community participation, and advocated using affordable, simple, so-called ‘appropriate’ technologies. It appeared as a distinct departure from sophisticated hospital-based health care for the privileged few and from the techno-centric, single-disease programmes that had dominated international public health efforts in the 1950s and 1960s.^3^ Importantly, primary health care also signalled a return to the doctrine of social medicine, which had featured significantly in medical debates in the 1930s and 1940s.^4^ Social medicine emphasized the broader social and economic context of health and criticized narrow, bio-medical and clinical understandings of disease. Proponents of social medicine were sceptical about sophisticated medical technology. They advocated broader sanitary interventions and improvements in housing and nutrition, preferring horizontal interventions that aim to improve general living conditions to top-down vertical programmes that target single diseases.^5^

Mahler appears as a pivotal figure in the history of global health in the second half of the twentieth century. He is remembered as a visionary and charismatic leader who often expressed controversial views on the politics of health.^6^ In this article, I understand the years Mahler spent in India as an important and formative episode in his life. In particular, I seek to trace and analyse how Mahler negotiated ideas associated with social medicine, which circulated in India during his posting there, and which appear connected to his positions in the 1970s. One should be cautious about claiming simple, immediate, and direct relations between Mahler’s experience in India in the 1950s and the views he advocated two decades later. Yet, the analysis presented here assumes that a review of how conditions and debates in India influenced Mahler in the early stages of his career contributes to a fuller understanding not only of Mahler himself but also of the debates on primary health care, in which he was a crucial stakeholder.

## From Copenhagen to New Delhi

Born in rural Denmark in 1923 as the son of an austere parish priest and a mother who came from a family of physicians, Mahler studied medicine at the University of Copenhagen and graduated in 1948. He went through a conventional, clinically oriented study programme. The regulations in force largely confined the public health-oriented dimensions to one course in hygiene over two semesters (one academic year) relatively late in the programme.^7^ The importance attached to this course was limited. In 1944 and 1945, it was conducted by an assistant, and when a newly appointed professor died in March 1948, it was suspended for a year.^8^ The official study guide even admonished students not to get out of touch with the main clinical subjects during the year they – almost regrettably – had to devote some time to the course in hygiene. It is perhaps indicative of the relatively neglected status of the social dimensions of medicine in Copenhagen that the textbook for the course was Norwegian (authored by the fairly well-known Carl Schiøtz and reported as currently unavailable in 1944).^9^

In 1945 – midway into his studies – Mahler received a grant that allowed him to move into the all-male student residency ‘Regensen’, situated in the centre of Copenhagen.^10^ The Social Democratic Party dominated post-war society in Denmark, but the residents at ‘Regensen’ – who mainly came from a middle-class background – tended to hold conservative and centre-right views.^11^ Within this community, Mahler was referred to as a person with socialist sympathies. It is not clear, however, whether he was a supporter of the moderate social democrats or he had sympathies with the more radical communists.^12^ The possibilities for a student of medicine with socialist views to engage in critical discussions on health and medicine outside formal education were few. In the 1930s, the group ‘Socialistisk Medicinergruppe’ (Socialist Medicals) – presumably inspired by *Mot Dag* and Karl Evang in Norway – conducted studies on connections between health, nutrition, and poverty and argued for the establishment of a university chair in social medicine. During the German occupation, members joined the resistance organization ‘Frit Danmark’ (Free Denmark), which was apolitical but dominated by communists. In 1947, this group presented a proposal for a revised study programme in medicine. It suggested moderately strengthening the position of hygiene and social medicine. However, its primary concern was to make medical education more relevant for the future general practitioner and therefore proposed more clinical and less theoretical teaching.^13^ A modest result of the proposal was that K. H. Backer – the editor of the national journal aimed at general practitioners – was hired to conduct a lecture series on the organisation of medical services, which took inspiration from lectures developed in Birmingham under Thomas McKeown.^14^ Although these debates and initiatives took place as Mahler finished his education, there is no evidence to suggest that he took an active part in them.

Mahler grew up in an emerging social democratic welfare state, where health increasingly became a government responsibility. Various state agencies conducted campaigns to ‘educate’ the population to adopt a healthy lifestyle and surveyed the population’s health status through systematic, large-scale examinations of school children. Access to health care for the large majority was secured through insurance-based sick-benefit associations. These associations had their roots in nineteenth-century local initiatives, but by 1945, they received significant public funding. Membership was mandatory for all adults, including those too wealthy to get benefits. This began to resemble a universal health care system, and debates began in the late 1940s – as Mahler finished medical school – about transforming the system into a tax-based, state-run health care system.^15^ Therefore, to a large extent, debates on social medicine in post-war Denmark were about ‘socialized medicine’ and the future status of the family doctor. Most commentators envisaged the transformation of the independent doctor into a state employee. Danish observers reported extensively from the United Kingdom on implementing health-related parts of the Beveridge Plan. In 1945, Backer’s journal published a positive assessment of Henry Sigerist’s ‘Socialized Medicine in the Soviet Union’.^16^ Mahler’s background combined, therefore, the experience of growing up in an emerging welfare state with a clinically oriented medical education. Although he held leftist political views, exposure to ideas associated with social medicine was limited.

After receiving his degree, Mahler appeared intent on leaving Denmark. In November 1949, he signed a contract with the International Tuberculosis Campaign (ITC), a Scandinavian initiative supported by the WHO and UNICEF to vaccinate against tuberculosis with the controversial BCG vaccine in war-torn Europe and to introduce it elsewhere.^17^ His first posting was in southern Germany, but Mahler wanted to travel further. In March 1950, he complained to the ITC headquarters that he had been neglected when ITC selected staff for overseas positions, and a few months later, he was posted to the ITC campaign in Ecuador.^18^ As ITC was dissolved during the spring of 1951 and its campaigns taken over and expanded by the WHO and UNICEF in direct collaboration with national governments worldwide, Mahler was hired as WHO medical officer to the biggest of them all: the campaign in India.

Arriving in India in the mid-twentieth century was often a shocking experience for Western health professionals. Large sections of the Indian population lived under miserable conditions, and improving their health appeared to be a stupendous task. John Grant of the Rockefeller Foundation, who came to India in 1939 after having spent much of the 1930s in China – another large and poor Asian country – summed up his experiences when he told a New York audience in 1945: ‘I knew the Asiatic Countries and all of them had features way ahead of India. Medically it is the most backward country in the world.’^19^ Mahler’s predecessors – the Scandinavian nurses and doctors who travelled to India with the ITC between 1949 and 1951 – expressed similar views. One wrote that most Indians lived in such ‘incredible poverty’ that the BCG vaccine could be nothing more than ‘a drop in the ocean’. Another noted that those arriving with romantic expectations were bound to be disappointed: ‘From the first moment you are facing the brutal reality of need and misery, which for a period of time is shattering to anyone.’^20^

Numbers confirm this dismal picture. In the 1940s, India’s crude death rate was twenty-seven per 1000 – more than twice the rate in England – infant mortality 182 per 1000 live births, and the life expectancy at birth a mere thirty-two years. More than half of the deaths in India were registered as ‘fevers’ – which meant mainly malaria – and it was assumed that tuberculosis killed at least 500 000 (or one in 600) annually.^21^ Mahler came from a country where people, on average, lived twice as long and died for entirely different reasons: cancer, heart disease, and conditions associated with old age. The mortality from tuberculosis in Denmark in the late 1930s, which at that time was a reasonable measurement of the general standard of living, was one in 2500. In 1951 – when Mahler arrived in India – it was further reduced to one in 7500.^22^

Like many of his colleagues, Mahler might have been shattered by the stark realities of disease and death in India as he transferred from prosperous Copenhagen – via Germany and Latin America – to poor Delhi. Yet, if this was the case, he did not express it in his official correspondence, which is virtually free of graphic descriptions of Indian misery. Importantly, in India, Mahler not only met deplorable health conditions and a vastly insufficient health service, he also encountered public debates on health and medicine, where ideas associated with social medicine were not only different from what he knew from Denmark but also much more pertinent.

## Social medicine: an Indian answer to health challenges

The foundational document for the debate on public health in post-independence India was the comprehensive report of the Health Survey and Development Committee, commonly known as the Bhore Committee. The Committee was established in 1943 with the double task of conducting an extensive survey of existing health services in British India and making recommendations for their future development. Its report was published in 1946 and appeared as a clear endorsement of the general doctrine of social medicine, as it had developed from the 1930s. The Committee defined health in a characteristically broad sense:

‘The term health implies more than the absence of sickness in the individual and indicates a stage of harmonious functioning of the body and mind in relation to his physical and social environment, so as to enable him to enjoy life to the fullest possible extent and to reach his maximum level of productive capacity’.^23^

Introducing the health plan for the future, the Committee made it clear that it gave priority to broad interventions in the environment:

‘At the outset, we must ensure the conditions essential for healthful living in town and country-side. Suitable housing, sanitary surroundings and a safe drinking water supply are the primary conditions for securing such a measure of environmental hygiene as is essential to ensure the pre-requisites of a healthy life.’^24^

A few pages later, the report explicitly referenced and aligned itself with social medicine. This approach to health had ‘widened the conception of disease from the narrow view of tissue changes and microbial and other specific causes by the inclusion of social, economic and environmental factors which play an equally important part in the production of sickness’.^25^ The report also cautioned against any belief in technological fixes. It declared that the desired ‘new health order’ could not ‘be achieved through a bottle of medicine or a surgical operation’ and saw ‘no magical wand to wave these changes into being overnight.’^26^ In the introduction to a chapter on environmental hygiene, this position was once again stated with admirable clarity: ‘In the campaign for improved health, drugs, vaccines and sera can in no way replace such essentials as a hygienic home, good food, fresh air and a safe water supply.’^27^

These views found support from India’s political leader. In 1950, Prime Minister Nehru attended The Third Health Minister’s Conference, where he delivered an address that turned out to be a genuine lecture on social medicine.^28^ From the state’s point of view, Nehru declared, individual treatment was ‘infinitely less important than the other important aspects, namely general public health, sanitation, hygiene, etc.’ Emphasizing the importance of food and housing, he voiced a poorly disguised criticism of the presumed clinical outlook of the Health Ministers of the Indian States: ‘These are not normally within your purview and probably your conference will not consider them.’^29^

Ideas associated with social medicine were not only promoted by Nehru, but they also dominated the official approach to health in the first five-year plan from 1951. Having established the fundamental importance of health to national progress, the plan defined it in terms similar to the Committee: ‘Health is a positive state of well-being in which the harmonious development of physical and mental capacities of the individual lead to the enjoyment of a rich and full life. It is not a negative state of mere absence of disease.’^30^ The plan then listed seven areas of priority. Malaria appeared as the only specific disease on the list, whereas all other priorities were broader interventions. Under the heading ‘Organisation and Administration’, the plan explicitly referred to the Bhore Committee and endorsed its view that establishing peripheral primary and secondary health units throughout the country was ‘of the greatest importance in providing broad based health services to the community.’ The plan took pride in the fact that the increase in expenditure for programmes categorized under ‘public health’ was much larger than for those under ‘medical’.^31^ Provision of a safe and adequate water supply and hygienic disposal of waste was accorded the highest priority and considered first, followed by nutrition, which was described as ‘perhaps the most important single factor in the maintenance of health and resistance to disease’.^32^ In July 1951 – when Mahler’s tenure as WHO Senior Medical Officer to the BCG campaign officially began – these views were put in circulation. A draft outline of the first plan was presented ‘for general discussion and comment’ and ‘for the widest possible public discussion.’^33^

## Combatting infectious disease

However, adherence to social medicine came under increasing pressure during the 1950s, as vertical campaigns based on the technological fixes, against which the Bhore Committee warned, became both available, due to technological breakthroughs and feasible, due to overseas assistance. A brief account of how the vertical campaign came to dominate the attempts to control malaria and tuberculosis – considered the two most deadly infectious diseases in mid-twentieth-century India – illustrates this transformation.^34^

The large-scale employment of the insecticide DDT against malaria was the quintessential technological fix in mid-twentieth-century disease control. Even the Bhore Committee found potential in its miraculous effect against malaria.^35^ Although the Committee emphasized the crucial need to establish a permanent and adequately staffed malaria organisation throughout India, it also considered DDT a valuable and highly effective remedy to be applied with the existing organic insecticide, pyrethrum.^36^ Five years later, DDT occupied centre stage in the first five-year plan. According to the plan, the use of the new insecticide had ‘brought about far-reaching changes in the technique of the control of malaria, and it has been successfully controlled with dividends several times the expenditure involved.’^37^ A similar enthusiasm was apparent in a lecture series delivered at the University of Madras in 1951 by one of the highest-ranking medical bureaucrats in India, the Director-General of Health Services, Dr. K. C. K. E. Raja. He found the results of initial DDT-based campaigns in Ceylon (Sri Lanka) and Bombay State ‘striking beyond measure’, and praised the simplicity of modern malaria control.^38^

India followed a global trend in its increasing enthusiasm for DDT. Given its meagre economic resources, India was eager to attract foreign assistance. A DDT-based programme against malaria offered an unequalled opportunity to obtain significant financial support, not least from the United States.^39^ Spurred by the Americans, the WHO launched its malaria eradication campaign in 1955, and the Union Minister of Health Rajkumari Amrit Kaur – who was deeply engaged in international collaboration and an important figure in the early years of the WHO – immediately impressed on the Central Council of Health that India had to join the eradication effort.^40^ The 1956 second five-year plan presented the malaria programme exclusively focusing on DDT spraying. It contended that getting malaria under control was possible before resistance would become a problem.^41^ By the middle of the 1950s, India believed that it had employed a ‘magic wand’ against its most dreaded infectious disease.

Tuberculosis was not only Mahler’s field; it also exemplified a social disease intimately tied to the conditions under which people lived. The Bhore Committee defined tuberculosis as such and argued that tuberculosis should be attacked through ‘improvement of the socio economic conditions so as to provide for the people a higher standard of living.’ However, the Committee deemed this to be beyond its field of enquiry, and concentrated on the ‘direct attack on the reservoirs of infection’.^42^ With an estimated 2.5 million infective cases of tuberculosis, just 6000 beds, and less than one-hundred tuberculosis specialists to care for them, it was out of the question to imitate the sophisticated hospital and sanatoria treatment known from Western countries. Instead, the Committee suggested ‘organized home treatment’ radiating from a grid of simple tuberculosis clinics as the centrepiece in the tuberculosis control strategy for a future India.^43^

The recommendations of the Bhore Committee concerning tuberculosis were issued without any references to drugs and vaccines. This was partly a question of timing. Streptomycin – the first antibiotic against tuberculosis – was discovered in 1943 but did not become generally available until after the report’s publication. The BCG vaccine, by contrast, had been around since the 1920s but did not feature in the report of the Bhore Committee. By early 1948, the government of India had decided to introduce BCG. Presumably, this sudden change was linked to the opportunity of getting foreign assistance. The ITC began vaccinating in Europe in 1947, and in November 1948, India signed an agreement with the campaign as the first country outside Europe.^44^

The paragraphs on tuberculosis in the first five-year plan reflected the speedy acceptance of BCG, which was now a new top priority in tuberculosis control. The plan found that BCG as a preventive measure yielded ‘the best return for the limited resources now available’ and expected successful mass vaccination over 15 to 20 years to reduce mortality from tuberculosis to a fifth. Moreover, UNICEF and the WHO had agreed to take over from ITC and provide financial and technical assistance to a countrywide mass vaccination drive. Mass BCG vaccination brought the vertical single-disease campaign to India; it also brought with it, Halfdan Mahler.^45^

BCG vaccination immediately provoked a forcefully articulated – if mainly regional – opposition. In Madras, the energetic retired sanitary engineer and editor of the magazine *People’s Health*, A. V. Raman, thundered against BCG. He could refer to shaky scientific evidence of the vaccine’s efficacy. Still, the main thrust of his argument was that BCG was a technological fix that would not solve India’s real health problems and betrayed the legacy of the Bhore Committee and its endorsement of social medicine. In February 1949, Raman clearly articulated the opposition between social medicine and vertical, techno-centric public health campaigns and even brought Gandhi into the picture:

‘We would give the first and foremost priority to the improvement of nutritional and environmental conditions. The Bhore Committee has said so. Long before the Bhore Committee, Mahatmaji said so. The fact, however, is that there is hardly a single health minister in any province today, who is troubled by the disparity between official policies and the Mahamaji’s teachings’.^46^

Raman’s opposition had died down before Mahler arrived in 1951. However, the Madras state was still foot-dragging about BCG under Chief Minister C. Rajagopalachari, a high-standing veteran of the nationalist movement and a close associate of Gandhi. Opposition flared up again in 1955, just as Mahler prepared to leave India. Rajagopalachari had resigned as Chief Minister of Madras and could freely voice his long-standing scepticism toward BCG. It differed from Raman’s; Rajagopalachari was less concerned with general medical policies and more concerned with the safety and efficacy of the vaccine, which he combined with increasing hostility toward the modernist and interventionist nature of the Indian state under Nehru’s leadership.^47^ The opposition to BCG – and Raman’s in particular – is significant in the present context because it posed social medicine *against* (some versions of) public health and must have impelled Mahler to reflect on the appropriateness of the campaign he was supervising.^48^

Despite the opposition from Madras, BCG vaccination remained the top priority against tuberculosis in the second five-year plan. Still, note was also taken of the potential of another of the ‘magic bullets’ of the 1950s: antibiotic drugs. The emergence from the 1940s of drugs effective against tuberculosis opened the possibility to have ‘a large number of tuberculosis patients treated in their homes’.^49^ By the late 1950s, the availability of both a vaccine and drugs against tuberculosis called for efforts to design a comprehensive tuberculosis control programme, that did not build on sophisticated and expensive institutions. Once again, Mahler was seconded to India on WHO duty. This time he went to the NTI in Bangalore, where he became one of the architects of India’s national programme to combat tuberculosis.

## Doctors, nurses, and auxiliaries

Views associated with social medicine inevitably questioned the privileged position of the highly skilled medical doctor. If the extension of health services was more important than their sophistication, educating more but lesser skilled and, therefore, cheaper types of health workers might be more appropriate. This issue divided the Bhore Committee. The majority resolved that India’s resources be ‘concentrated on the production of only one and that the most highly trained doctor, which we have termed the “basic” doctor’. The basic doctor was to hold a five-year university degree, which must include community, preventive, and ultimately social medicine: ‘Preventive medicine leads easily to social medicine, and it is as exponent of the principle of social medicine that we would wish the “basic” doctor to go forth into the world of medicine.’^50^ A consequence of promoting the basic doctor was abolishing the class of less-educated licentiates, to which two-thirds of the exiting cadre of Indian doctors belonged.

A minority of six members disagreed with this position. According to their minute of dissent, the shortage of doctors in India – particularly in rural areas – meant that the most pressing issue was to ‘increase their numbers to the maximum extent in the minimum time’. In such circumstances, it would be a mistake to abolish the licentiate doctors, which could be adequately trained in three and a half years. They criticized the majority for blindly following the the contemporary British Goodenough Committee. At the same time, they took their lead from the more relevant Soviet experience, where semi-skilled ‘feldshers’ had proved highly valuable in the successful expansion of medical services in the Soviet Union. The minority view was overruled, and in a lofty tone, the Committee stated its belief in the highly skilled ‘physician of tomorrow’ as someone who would ‘naturally be concerned with the promotion of the new era of social medicine’.^51^

If India lacked doctors, it lacked nurses even more. The Committee’s estimate that India needed a long-term fivefold increase in the number of doctors was dwarfed by the need to expand the number of nurses almost one hundred times: the existing number of 7750 was to grow into 680 000! To meet this challenge, the Committee did not hesitate to recommend – in contrast to its position on doctors – that there should be two grades of nurses.^52^ Considering the severe shortage of qualified doctors and nurses, the Committee recognized the need to employ semi-skilled health assistants; however, this type of health worker did not command much enthusiasm in the report. Although necessary ‘to relieve the medical man of many of his minor duties’, the health assistant was seen as a short-term solution. The Committee doubted that ‘in the larger scheme under the long-term programme, there will be room for a man of such limited technical skill’. Although use of less-qualified personnel – be it ‘feldsher-type’ doctors or health assistants – was sometimes associated with social medicine, the Bhore Committee largely repudiated their use in India. Only highly qualified personnel should serve the population in a future, independent India.^53^

Raja’s lectures also touched on the relationship between the skilled doctor and the semi-skilled auxiliary. Before rising to his high governmental post, Raja had served as Secretary to the Bhore Committee, and it is not surprising that his views followed those of its report. If Raja seemed optimistic about employing less-skilled workers and recommended training of ‘a large body of non-medical preventive health workers’, he remained deferent toward the highly skilled doctor. He duly explained that his emphasis on ancillary staff was ‘not intended to minimize in any way the importance of medical men in the health organization’. To the contrary, auxiliaries were necessary to ensure that doctors spent their valuable time on ‘more important jobs’.^54^ If the doctrine of social medicine inspired the official Indian approach to health, adherence to the highly skilled doctor remained a central tenet in it.

## Community participation and the indigenous practitioner

Although better and properly educated medical servants would bring health services to the population, the Bhore Committee also emphasized the importance of the populations’ active participation. The people had to be ‘aroused from their apathy’ before the prevailing deplorable level of sickness could be overcome:

‘A spirit of self-help should be created among the people through the development of co-operative effort for the purpose of promoting curative and preventive health work. In the programme of health development, which we put forward, the need for securing the active co-operation of the people in the day to day functioning of the health organisation should be prominently kept in view.’^55^

However, a related and more contested issue was whether this urge to secure community participation extended to the incorporation of indigenous traditions of medicine. The Bhore Committee was adversarial. It accepted that indigenous medicine was influential, cheap, and might contain ‘empirical knowledge’ of some value; but also warned against promoting these traditions for ‘patriotic pride’. This was a reference to attempts to position indigenous traditions of medicine as a marker of pride and cultural difference in the nationalist movement. The Committee had little time for such sentiment: ‘We do, however, say quite definitely that there are certain aspects of health protection which, in our opinion can be secured, wholly or at any rate largely, only through the scientific system of medicine.’ To comply with national pride, it added that modern scientific medicine was neither Eastern nor Western but a ‘corpus of scientific knowledge and practice belonging to the whole world and to which every country has made its contribution.’^56^ Therefore, the basic doctor suggested by the Committee was unambiguously trained in Western medicine, and there was no dissent on this issue.^57^

However, one indigenous group had to be incorporated into the future health services for ‘many years to come’. That was the Indian midwife, the *dai.* The maternal and infantile death rates were described as ‘apalling’, and the number of midwives in British India had to rise from 5000 to 100 000. If the inclusion of the Indian *dai* was not desirable, it was inevitable. The Committee described the *dai* without enthusiasm, expecting that ‘the dead weight of ancestral tradition may be so heavy on her’ that her successful incorporation into the health services would be difficult. The *dai* was imagined as a figure that had to be ‘won over’ for scientific medicine, and the Committee advised to proceed ‘only by stages and with a sympathetic understanding of her own background of ignorance and prejudice, to win her over to the adoption of certain necessary changes in her traditional practice.’^58^ Although these views on the *dai* neatly condense the paternalistic attitude towards indigenous medicine represented by the Bhore Committee, they also pointed to the significant and necessary resources of manpower that could be tapped from indigenous traditions.

At the Health Ministers conference in 1950, the endorsement of indigenous traditions of medicine was high on the agenda. Nehru was sceptical, but attempted to be modern and ‘Indian’ at the same time. Although he did not find that indigenous medicine paid much attention to public health or sanitation, he denounced – as an echo of the Bhore Committee – the expression ‘Western medicine’. Modern medicine was, he said, ‘as much Eastern as it is Western’ because it was scientific. To his mind, there was ‘no doubt at all that the Ayurvedic and the Unani systems have excellent remedies’ and that they should be integrated easily into scientific medicine. Nehru insisted, however, ‘that adequate training in modern medicine should be given to every medical practitioner.’ After receiving this training, Nehru accepted that the doctor was free to practice within indigenous traditions of medicine.^59^

Kaur also addressed the Minister’s conference on this issue, and she stroke a somewhat different chord. She went straight to a severe and undisguised criticism of indigenous medicine, which she associated with ‘quackery’. Kaur reminded her audience of all the crucial aspects of medicine, which Ayurveda and Unani lacked: ‘In the circumstances, it is unimaginable that India can deliberately accept the indigenous systems of medicine as the bases on which to build her health services and ignore the claims of modern medicine.’^60^ While the genuinely modernist Nehru tried to show cautious respect for India’s medical traditions, Kaur was openly hostile. Ayurveda and Unani might have had a glorious distant past but were now ‘outmoded’ by the ‘incomparably superior’ modern medicine. It would, Kaur underlined, ‘be foolish and even criminal for India to decide that her health services should be built on any other foundation than that of modern medicine.’ It was, therefore, evident to Kaur that India could not afford to educate doctors with different qualifications than ‘the advanced countries of the world’.^61^

In his lecture series, Raja repeated the scepticism toward indigenous medicine expressed by the Bhore Committee, Nehru, and Kaur. He acknowledged that supporters of indigenous medicine ‘genuinely felt’ that these traditions represented a ‘rich heritage of medical knowledge’, but found that the government could not afford to ‘fritter away’ its limited funds on several systems of medicine.^62^ Raja accepted that indigenous medicine might contain valuable elements, and he argued that these should be incorporated into modern medicine if they were able to meet the requirements of modern science.^63^

Nationalist pride and genuine belief in indigenous medicine proved challenging to combat. The Health Ministers Conference ignored the admonitions of their political leaders. It passed a resolution, which proposed that courses on indigenous medicine be offered in at least one medical college in each state and that candidates fully trained in indigenous medicine should be employable by the state health services at salaries similar to those trained in modern medicine.^64^ Indigenous medicine also entered the five-year plans, although ambiguously. The first plan noted that a ‘great deal of uncertainty’ existed regarding the position of indigenous systems of medicine, homeopathy, and nature cure. Still, it emphasized this uncertainty had to ‘be cleared up as early as possible’. It allocated funds to set up a central research institute in indigenous systems of medicine. It impressed that there was ‘a large scope for research in order to improve and enlarge their special contribution to medical science.’^65^ The second plan, by contrast, dealt with indigenous medicine in one brief paragraph and simply noted that modest funds were allocated for the expansion of ayurvedic colleges and dispensaries. The former should be brought up to a standard enabling them ‘to take up research programmes’.^66^ Despite profound reservations from its modernist political and medical establishment, the Indian state accepted indigenous systems of medicine, but simultaneously required that the standards of modern science judged them.

## Mahler between public health and social medicine

Mahler’s work in India spanned a decade characterized by these lively debates on health policy issues, and he must have been well acquainted with them. The report of the Bhore Committee surely was compulsory reading for any doctor on long-term WHO duty in India, and Mahler presumably consulted the five-year plans, followed newspaper debates, listened to radio broadcasts, and indeed conducted numerous formative conversations with Indian colleagues.^67^ He also expressed his views on health and health policy issues in his official correspondence with superiors in Geneva and New York.^68^

A remarkable feature of Mahler’s early reports from the BCG campaign was a sharp anti-doctor rhetoric. Despite India’s formal allegiance to social medicine, Mahler deplored the prevailing clinical approach to medicine. In a speech delivered at a conference on BCG in Delhi in December 1952, he stated that he had ‘no doubt that doctors with a clinical background will never put up with the hard and monotonous BCG work’, and he, therefore, suggested that only young doctors ‘with a public health outlook’ should be recruited for this service. Mahler held that if the team doctor took no active interest in the BCG work, ‘it might be preferable to let such a team work without a doctor.’^69^ In a report from 1953, he addressed the self-styled condition of ‘BCG Fedupness’. He explained to his superiors that the cause of this ‘psycho-economic disease’ found in many public health programmes was ‘the clinical atmosphere pervading the Health Services in many states.’^70^ In an extensive, final report on BCG from 1955, he similarly complained about the ‘dearth of public health-minded doctors in a clinically infested atmosphere’.^71^ Mahler did concede that low salaries might explain the lack of enthusiasm for BCG work among highly qualified doctors. Still, his reports reveal a more profound aversion toward this group, which he referred to as over- or even super-dignified and with ‘a high-brow indifference to such a simple thing as reading a tuberculin reaction accurately and uniformally.’^72^ In the final report, he claimed that half of the qualified team leaders had ‘a positively harmful influence’ on the campaign. By contrast, the less skilled technicians was portrayed as ‘young’, ‘energetic’, and ‘fine boys’.^73^ This group, unspoiled and unaffected by the attitude of the clinically trained doctor, might be able to provide better health to India’s rural masses. He found it ‘pleasantly amusing, though a blow to most doctors’ professional conceit, that in India non-medical auxiliaries, after thorough training in a practical public health measure, do a better and more conscientious job than doctors.’^74^ Mahler’s aversion toward the doctor resonated with the views of the influential Norwegian health bureaucrat, Karl Evang, who travelled to India on a WHO mission in 1953. As Sunniva Engh has shown, Evang – a long-standing and outspoken supporter of social medicine – criticized what he perceived as India’s ‘strong emphasis for curative medicine’ and advocated a ‘fundamental reorientation’ from curative to preventive medicine.^75^ While Mahler thundered against the ‘clinical atmosphere’, Evang’s culprit was curative medicine. Common to them was a scepticism towards sophisticated medicine for the privileged few.

Another recurrent feature of Mahler’s reports on BCG was references to the potential of the mass vaccination campaign to educate the rural population on health issues and thus ensure community participation. Mahler might have found the health outlook of the Indian villager ‘passive’, but he soon began to emphasize the educative value of mass vaccination, which ‘will make it possible for us to give the rural public a clear idea of what modern public health can do for them.’ He ended an early report emphasizing the untapped resources of the masses:

‘Our results from the mass BCG campaign may still be deficient as regards quality and quantity but they irrefutably prove that the active interests of the masses in their own health is easily roused when an enthusiastic approach is made … (…) … The greatest asset, however, of this campaign may be the elightment [sic] of the rural masses in respect of the possibilities for achieving and the benefits derived from physical well-being. When first the rural masses realize and are able to formulate their demands for a healthier life, no power can deny it to them.’^76^

By 1954, Mahler stated that the campaign was ‘insisting on the imperative need for active cooperation of the public’ and identified one of its guiding principles as gaining the ‘confidence and active cooperation of the villagers through kindness and smile.’^77^ In his final report a year later, he defined the campaign’s purpose as both protection through vaccination and ‘health education’. He further claimed that the campaign was ‘the very first mass public health project which has to count on the enthusiastic cooperation by the public’. He emphasized how campaign workers took the villagers into confidence: ‘if successful this approach could have the greatest social impact on the villagers’ attitude towards outside help, and a healthy effect on the existing stagnated public health concept.’^78^

During his tenure as the WHO medical officer to the BCG vaccination campaign, Mahler appears to have seen himself as a combatant in a contest between ‘clinical medicine’ and ‘public health’. In characteristically vivid style, he complained about the reigning clinical outlook in the medical profession:

‘As long as it is considered infinitely more important to remove an inflamed appendix – occurring amongst the privileged 2 to 3 per cent of the total population – than to give 10 000 BCG vaccinations in the rural areas, one could hardly expect Public Health workers to develop that high working spirit which is indispensable for a high quality and sound economy of any Public Health programme in India.’^79^

Mahler believed, however, that the vaccination campaign would have a profound impact. According to one report, it had ‘greatly catalysed the process of converting the minds of medical people from looking clinically at tuberculosis to conceiving the disease as a public health problem.’^80^ According to the final report on BCG, Mahler emphasized that vaccination was ‘a public health measure, adopted against a public health problem’, and claimed that it had dealt an ‘urgently needed blow’ to the clinical approach to tuberculosis. In the broader sense, the ‘mass BCG project has through mobilizing a whole-hearted cooperation and understanding of the public catalized [sic] the process creating demands for more public health.’^81^

If public health served as the good antidote to the faulty clinical medicine in Mahler’s reports up to 1955, he did engage with the fundamental assumptions in social medicine in his final report on BCG. He began by playing down the importance of specific infectious diseases – cholera, plague, malaria, and even tuberculosis – when contrasted to more fundamental problems such as malnutrition, mental disease, and widespread gastrointestinal conditions: ‘The greatest difficulty in the promotion of health is probably the lack of genuine understanding among doctors of the *social impact* of public health conditions.’^82^ He noted that environmental sanitation was ‘at a very low ebb in India’ and that the congestion in urban slums was extreme: ‘Too few can afford an adequate intake of calories, and less would take trouble at a balanced diet.’^83^ Mahler even admitted that the effect of BCG vaccination and other specific measures taken against tuberculosis had never been ‘established beyond doubt’ and indicated that raising the general standard of living mattered: better housing, better nutrition, and general environmental sanitation.^84^ This was a new accentuation in Mahler’s views, and it came much closer to the core assumptions of social medicine than anything he had written in his first four years in India.

## Toward community-oriented medicine

When Mahler returned to India as Senior Medical Officer seconded to the NTI in Bangalore, his job was broader than supervising and managing a vertical mass vaccination campaign. The task for Mahler and his colleagues at the NTI was to develop a model for a tuberculosis control programme that was both effective, applicable, and affordable under Indian conditions.^85^ Two interesting early reports presented the ‘outlook’ of the institute, and although the author(s) of these reports are not given, it is inconceivable that Mahler was not involved.^86^ One of the reports defined the objectives of the NTI as being to ‘reduce India’s tuberculosis problem, gradually but as quickly as possible, within the funds judged to be available for the purpose, and making maximum use of and developing general health services.’^87^ This definition suggested both cost-effectiveness and a preference for a horizontal and integrated approach.

The early reports unequivocally stated that prevention is better than cure. Inspired by the Swedish development economist Gunnar Myrdal, health investment was favourably contrasted to health consumption and expensive institutional treatment of tuberculosis patients written off as ‘conspicuous consumption’.^88^ The view on the vertical nature of preventive campaigns – such as mass BCG vaccination – was more ambiguous. One of the reports emphasised that there was ‘a strong a priori case against specialized service’, and further explained

‘At a time where the whole policy of the country is directed towards the establishment of a skeleton generalized health service, ultimately to be developed to bringing health services to the farthest villages, all moves to divert money, personnel and equipment to other purposes must be viewed with the greatest anxiety … (…) … Only if the specialist agrees to build in their specialities into the national health service can this service become strong enough to meet its task, and only through such integration can the special services expect to be brought out to the masses of the population.’^89^

Despite this and other references to the desirability of integration with the general health services, the ongoing – and paradigmatically vertical – DDT-based malaria eradication effort was referred to as successful and a preventive ‘investment’ yielding an immediate return. The return from the equally vertical BCG vaccination was more distant. Still, the beauty of BCG was that – thanks to its operational simplicity – it was suitable for an initial specialized campaign and later integration.^90^ An elaborate mathematical modelling of three approaches in tuberculosis control – one based on mass vaccination and two based on different types of case-finding and drug treatment – concluded that mass BCG vaccination was the most cost-effective investment India could make in its attempt to control tuberculosis.^91^ By 1959, Mahler remained supportive of the vertical campaign that had brought him to India.

Mass BCG vaccination was a techno-centric enterprise, and the approach of the NTI toward drugs and vaccines was generally positive as long as they were simple, affordable, and readily available. One of the reports noted, for instance, that combined with expected improvements in living standards, the potentialities of BCG vaccination and cheap and effective drugs made it ‘not unrealistic that India’s professed aim in the field of tuberculosis should be the elimination of the disease as a public health problem within the next twenty years.’^92^ Although the other report expressed some reservations against a drug-based strategy – it was too expensive, difficult to integrate in the general health services, and could accelerate the development of drug resistance – it also recognized ‘that the world’s trend and India’s trend, is towards mass application of drug treatment.’^93^ In 1946, the Bhore Committee warned against believing too much in drugs and vaccines. Well over a decade later, Mahler and the staff at NTI were much less sceptical. Borrowing a term from the debates in the 1970s, they found these technologies ‘appropriate’.

In his reports from the BCG campaign, Mahler emphasized the desirability to secure the active cooperation of the population. This view also surfaced in the NTI reports, where tuberculosis control became linked to community development. Community development was one of the top priorities in the second five-year plan. It was – particularly for rural programmes – of ‘immediate practical importance to study how a tuberculosis control programme can be implemented through the machinery of community development projects.’^94^ Moreover:

“The decisive factor in the success of community development is the mobilisation of the villagers for active individual and collective participation in the programme. The anti-tuberculosis work is to adopt this approach. The programme will be operated through existing and potential social patterns, institutions and public servants”.^95^

When Mahler left India in early 1961, the community-oriented approach had been consolidated at NTI. A ‘Prospectus’ noted that although tuberculosis had hitherto been approached from the (clinical) point of the individual patient and control in the community at large therefore neglected, the measures applied by the Institute would attempt ‘to make use of the available and existing community organizations associated with [the] public health department and agencies such as Community Development.’^96^ Several of the early studies conducted by the NTI would consider both a mass campaign approach and a community development approach.^97^

The emphasis on community development and participation further developed into an awareness of the need to see tuberculosis from the patient’s perspective. The actual measurement of India’s tuberculosis problem was not the abstract symbolic representation in numbers and statistics but ‘the felt problem’. Sociology became as important as epidemiology in determining how to deal with tuberculosis. Among the earliest studies from the NTI were groundbreaking, sociologically oriented studies of patient awareness of symptoms and the acceptability of proposed drug regimens.^98^

This perspective suggests the profound influence of the Indian doctor Debabar Banerji, who Mahler hired in 1959 as a sociologist at the NTI. In his autobiography, Banerji retrospectively identifies the central purpose of his life as ‘subordinating medical knowledge to the people, rather than the other way around’. He also reveals how he developed a cordial and lifelong friendship with Mahler and his international sociologist counterpart at NTI, Stig Andersen.^99^ Later in life, Mahler acknowledged that he learned much from Banerji and in 2007 emailed him declaring, ‘You still have many years to bless your pupils – and I am proud to be one these …’.^100^ In evening conversations in Bangalore, Banerji and Mahler might also have recalled the general vision advocated by the Bhore Committee, which Banerji eagerly and explicitly adopted. In an article from 1962, he lamented the prioritisation of expensive health institutions during colonial rule – tuberculosis sanatoria would be the paradigmatic example – and found the achievements of post-colonial India equally flawed by the influence of ‘foreign trained technological crusaders’. Banerji saw extreme poverty as the root cause for India’s health problems, and called for plans ‘for having better nutrition, better water supply and housing and better education …’.^101^ This was one of several echoes of India’s vision of social medicine that Mahler heard during his time in India.

## An additional path to Alma-Ata

Twelve years after leaving India, Mahler was elected Director-General of the WHO and became one of the architects behind primary health care as a strategy to reach the vision of ‘Health for All’. Primary health care was inspired by a wide range of experiences and ideas ranging from the failure of the vertical, DDT-based malaria eradication programme to the basic needs approach to development and communist China’s barefoot doctors. It also contained significant affinities with the doctrine of social medicine, as it developed in the 1930s and 1940s.

Although Mahler was neither the principal author of the central texts expounding primary health care nor its chief ideologue, he was a crucial stakeholder. The zeal with which he promoted primary health care and how he framed it in his captivating speeches were important for its realization. It is, therefore, appropriate to ask where Mahler’s strong sympathy and tireless support for primary health care came from. WHO staff, who worked with Mahler as Director-General, suggested his experience in India was crucial. Socrates Litsios, who was part of the group working with primary health care in the 1970s, has claimed that much of Mahler’s thinking ‘can be traced back to his experiences in India.’^102^ Daniel Tarantola, who worked with Mahler in the 1980s, agrees:

‘I am totally convinced, that his philosophy came from India. … (…) … You are exposed to inequalities and inequities in such a way that you can’t cope with it. Mahler referred often to his Indian exposure to poverty and inequality. I do not think he ever had a guru, but he must have listened to lots of wise Indian interlocutors who created that consciousness’.^103^

The preceding analysis elaborates on these suggestions and proposes how views formed and lessons learned by Mahler while in India might have informed his contributions to the debates on primary health care two decades later.

In 1951, Mahler came to a country where existing health services were inadequate, and the new state faced stupendous challenges in improving the health of its massive population. He also went to a country that endorsed the doctrine of social medicine as the most appropriate strategy to improve the situation, even if its influence faded during the 1950s and the general atmosphere remained ‘clinical’. The evidence presented here suggests that Mahler grew increasingly sympathetic toward social medicine while he was in India. A salient feature of Mahler’s views was a remarkable aversion to the clinically trained and highly skilled doctor. From the beginning of his term as medical officer to the BCG campaign, he found them unsuitable for public health service but praised the work of semi-skilled auxiliaries. Although these views differed from the prevailing confidence in highly skilled doctors in India in the 1950s, they are recognizable in the later endorsement of community health workers within primary health care. Mahler also encountered and embraced the notion that active cooperation of the population in health issues is crucial. Towards the end of his service in India, he was developing a tuberculosis control programme that was both community-oriented and patient-centred. Twenty years later, community participation became one of the crucial elements in primary health care. Finally, Mahler witnessed how strong and resilient indigenous medicine traditions were not only seen as crucial suppliers of manpower in the form of *dais*, but also withstood hostility from the modernist top figures in Indian health politics. Although Mahler did not comment on the value of indigenous traditions of medicine during his stay in India, it is notable that primary health care identified practitioners of indigenous medicine as an untapped health resource.

## Concluding remarks

There can be little doubt that Mahler’s decade-long experience in India significantly formed his perspective on health. He arrived in India with a conventional clinical education, leftist political sympathies, modest exposure to ideas related to social medicine, and as the representative of a vertical, top-down public health campaign. He left the country a decade later sympathetic toward ideas associated with social medicine, hostile toward the highly skilled doctor, convinced that sophisticated technology and expensive institutions were superfluous – but community participation crucial – to promoting health in developing countries. The experience in India not only formed Mahler as a person but also help explain why he became such an ardent and convincing advocate of primary health care two decades later. This does not mean that the views Mahler formed in India in the 1950s were transferred directly into the debates on primary health care in the 1970s. Nor does it mean that Mahler was the grand inspiratory architect of primary health care. More modestly, it suggests that Mahler was one of the vehicles that connected ideas associated with social medicine as it developed from the 1930s – and appeared in India in the 1950s – with the vision of primary health care in the 1970s.

## Footnotes

2 ‘Mahler, Halfdan Theodor’, *Den danske lægestand 1965–82*, II (Copenhagen: DADL, 1982); ‘Dr. H. Mahler’, Biographical Note, WHO Archives, Geneva; See also Tine Hanrieder, ‘Mahler, Halfdan Theodor’, https://www.ru.nl/politicologie/io-bio/io-bio-biographical-dictionary-sgs-ios/. Accessed 15 June 2022.

3 The content of primary health care is expounded in *Primary Health Care. A Joint Report by the Director-General of the World Health Organization and the Executive Director of the United Nation’s Children’s Fund* (Geneva & New York: WHO and UNICEF, 1978). For a ‘distillation’ of the principles behind it, see Niels Brimnes, ‘Bandung Revisited: from rural hygiene to primary health care’ https://projects.au.dk/inventingbureaucracy/blog/show/artikel/bandung-revisited-from-rural-hygiene-to-primary-health-care/. Accessed 23 September 2021.

4 Randall Packard has described primary health care as ‘a radical departure from the culture of technical assistance’ and as ‘a return to the earlier vision of health and development’, which characterized the League of Nations Health Organization and the Rockefeller Foundation up to World War II. Randall Packard , A History of Global Health. Interventions into the Lives of Other Peoples (Baltimore: Johns Hopkins University Press), 227–28. See also Brimnes, *ibid.* and Mahler’s own invocation of the continuities between primary health care and rural hygiene in Halfdan Mahler, ‘Promotion of Primary Health Care in Member countries of WHO’, *Public Health Reports*, 93, 2 (1978), 107–13.

5

Sunil Amrith , Decolonizing International Health. India and Southeast Asia, 1930–65 (Basingstoke: Palgrave MacMillan, 2006), 29–42 . Iris Borowy, *Coming to Terms with World Health. The League of Nations Health Organization 1921–46* (Frankfurt: Peter Lang, 2009), 199–204, 325–60. Packard, *op. cit.* (note 4), 47–88.

6

Socrates Litsios , ‘The Long and Difficult Road to Alma Ata: A Personal Reflection’, International Journal of Health Services, 32, 4 (2002), 716–1810.2190/RP8C-L5UB-4RAF-NRH212456122. Marcos Cueto , ‘The Origins of Primary Health Care and Selective Primary Health Care’, American Journal of Public Health, 94, 11 (2004)1551422110.2105/ajph.94.11.1864PMC1448553. Nitsan Chorev , The World Health Organization between North and South (Ithaca: Cornell University Press, 2012), 55–59 . Hanrieder, *op.cit.* (note 2).

7 ‘Kongelig Anordning af 25. September 1936 med senere Ændring af 25. August 1941 om den lægevidenskabelige Embedseksamen ved Københavns Universitet’ and ’Vejledning til Studiet’, both in *Regler for samt Studieplan over Fagene til den lægevidenskabelige Embedseksamen* (København: S. L. Møllers Bogtrykkeri 1944).

8
*Forelæsninger og Øvelser ved Københavns Universitet, Aarhus Universitet og den Polytekniske Læreanstalt, 1941–45 and 1946–50* (Copenhagen: S. L. Møllers Bogtrykkeri).

9 ‘Vejledning til Studiet’, *op. cit* (note 7), 26 and ‘Lærebøger’, *op. cit.* (note 7), 29.

10

Stuart Ward (ed.), Regensen - bag de teglrøde mure (Copenhagen: Gyldendal, 2017).

11 The political sympathies of the residents is known from mock elections carried out simultaneously with general elections in 1943, 1945, and 1947. ‘Protokol 1942–45’, entry from 22. March 1943. 2405–12, Københavns Universitet, Regensen, Klokkeren; ‘Protokol 1945–48’, entry from October 1947. 2405–13, Københavns Universitet, Regensen, Klokkeren. Both in Danish National Archives.

12 ‘Protokol 1945–48’, entries from 8. March 1946, 2. September 1947, and around 1. December 1946, *ibid.*

13

Morten Thing , Kommunismens Kultur. DKP og de intellektuelle (Copenhagen: Tiderne Skifter, 1992), I, 439–45; *Forslag til Ny Lægevidenskabelig Studieordning. Udgivet af Frit Danmarks Lægegruppe* (Copenhagen 1947); ‘Diskussion om Studieplanen’, *Stud. Med.*, 1947, 4, 95–99.

14 K. H. Backer, ‘Praktiserende Lægers Opgaver og Arbejdsmetoder’, *Stud. Med.*, 1948, 1, 13–15; K. H. Backer, ‘Undervisning i Social Medicin ved Universitetet i Birmingham’, *Maanedsskrift for Praktisk Lægegerning og Social Medicin*, 1948, 10, 430–33.

15

Signild Vallgårda , Folkesundhed som Politik. Danmark og Sverige fra 1930 til i dag (Aarhus: Aarhus Universitetsforlag, 2003), 28–118 ; Jørn Henrik Petersen, Klaus Petersen, Niels Finn Christiansen, *Velfærdstaten i Støbeskeen: perioden 1933–56*, *Dansk Velfærdshistorie* vol. III (Odense: Syddansk Universitetsforlag 2012), 329–78.

16

Karl Teilmann , ‘Lægestandens Socialisering’, Maanedsskrift for Praktisk Lægegerning og Social Medicin, 1945, 3, 78–88 .

17 For an introduction to the International Tuberculosis Campaign, see Niels Brimnes , ‘Vikings against Tuberculosis: The International Tuberculosis Campaign in India, 1948-1951’, Bulletin of the History of Medicine, 81, 2 (2007), 407–301784472210.1353/bhm.2007.0022.

18 A. Breidal (Copenhagen) to H. Mahler (Munich) and ‘anonymous’ to H. Mahler (Munich), 20. March 1950, private papers in possession of Per Bo Mahler.

19 Quoted from Amrith, *op. cit* (note 5), 60.

20 Svendsen, Svend K., ‘Indiens Svøbe – Tuberkulosen’, *Flensborg Avis,* 6 December 1949; 27 og Breidal, Annalise, ’ITC-Læge i Indien’, *ITC Måneds-Nyt,* April 1950.

21

Roger Jeffery, The Politics of Health in India (Berkeley: University of California Press, 1988), 119–22; *Report of the Health Survey and Development Committee* (Calcutta: Government of India Press, 1946), vol. I, 7–8, 13.

22 Life expectancy, see https://www.statistikbanken.dk/HISB7. Accessed 24 September 2021. *Causes of Death in the Kingdom of Denmark, 1953*, Copenhagen: National Health Service, 1954, table 9, 8–9.

23
*Report*, *op.cit.* (note 21), I, 7

24
*Report*, *op.cit.* (note 21), II, 2.

25
*Report*, *op.cit.* (note 21), II, 7

26
*Report*, *op.cit.* (note 21), II, 3–4.

27
*Report*, *op.cit.* (note 21), II, 218

28 The report of the Bhore Committee was one obvious source of inspiration for Nehru, but so was the report on public health by the Congress’ ‘National Planning Committee’, which held similar and some cases more radical views influenced by social medicine. See Jeffery, *op. cit.* (note 21), 113; Amrith, *op. cit.* (note 5), 44–45.

29 Jawaharlal Nehru, ‘The Modern System of Medicine’, in *Jawaharlal Nehru’s Speeches*, (Delhi: Government of India, 1954), vol. II, 536–45, quoted from pp. 537 and 540.

30 Government of India, Planning Commission, *First Five Year Plan*, Chapter 32, Health, para 2, https://niti.gov.in/planningcommission.gov.in/docs/plans/planrel/fiveyr/welcome.html. Accessed 24 September 2021. This passage is also strikingly close to the first paragraph in the 1948 Constitution of the WHO, which refers to health as “not merely the absence of disease or infirmity”.

31
*ibid.*, Chapter 32, paras 8, 9 and 12, quoted from para 9.

32
*ibid.*, Chapter 32, paras 12, 15-31, quoted from para 23.

33
*ibid.*, Introduction, para 2.

34 Much of this section is based on a fuller version of the argument in Niels Brimnes, ‘Rallying around the Magic Wand: Visions of social medicine, public health and disease control in India 1946–1957’, *Health and Development. Yearbook for the history of Global Development*, vol. II (Oldenbourg: De Gruyter, 2023), 175-99. The move away from the doctrine of social medicine was not a uniform, one-way process, but a trend. In her contribution to this issue, Sunniva Engh has, for instance, identified continuing efforts to promote social medicine in educational programmes in India in the 1950s. See Sunniva Engh, ‘The complexities of postcolonial international health: Karl Evang in India 1953’, *Medical History*, XX, X (2023), xx–xx.

35
*Report, op. cit.* (note 21), I, 93, 96–97; II, 144, 147-8. Quotes from pp. 144 and 148

36
*Report, op. cit.* (note 21), II, 146.

37 Government of India, *op. cit.* (note 30), Chapter 32, para 33 and 34.

38 K. C. K. E. Raja, *The Building of the Nation’s Health*, *The Dr. Lakshmanaswami Mudaliar Endowment Lectures* (Madras: University of Madras, 1951), 8.

39 From 1952, the United States provided half the funding of the malaria program. Between 1950 and 1973 public sector assistance from the United States to anti-malaria programs were more than ten times higher than to water supply. Jeffery, *op. cit.* (note 21), 194–200.

40 Rajkumari Amrit Kaur, ‘On the Role of the Central Council of Health’, in G. Borkar (ed.), *Selected Speeches and Writings of Rajkumari Amrit Kaur* (New Delhi: Archer Publications, 1961), 231–50, quoted from pp. 240–41. On the malaria campaign and the influence of the United States in the WHO, see Marcos Cueto , Theodore M. Brown and Elizabeth Fee , The World Health Organization. A History (Cambridge: Cambridge University Press 2019), 86–107.

41 Government of India, Planning Commission, *Second Five Year Plan*, Chapter 25, Health, paras 32–34 https://niti.gov.in/planningcommission.gov.in/docs/plans/planrel/fiveyr/welcome.html. Accessed 27 September 2021.

42
*Report, op. cit.* (note 21) II, 158. See also I, 103.

43
*Report, op. cit.* (note 21), II, 159–64, quoted from p. 164. Similar warning against costly buildings were raised in the context of malaria. See II, 150.

44 Niels Brimnes, *Languished Hopes. Tuberculosis, the State, and International Assistance in Twentieth-century India* (Delhi: Orient BlackSwan, 2016), 81–4, 106–8.

45 Government of India, *op. cit.* (note 30), Chapter 32, paras 36–43. Quoted from para 37.

46
*People’s Health* III, 5 (1949), 203–4. Raman served at the national Environmental Hygiene Committee, which submitted its report in October 1949. See: https://www.indianculture.gov.in/report-environmental-hygiene-committee-1949-0 (accessed 27 September 2021).

47 For analyses of opposition towards BCG, see Sunil S. Amrith, *op. cit.* (note 5), 137–46; Christian W. McMillen and Niels Brimnes, ‘Medical Modernization and Medical Nationalism: Resistance to Mass Tuberculosis Vaccination in Postcolonial India, 1948–1955’, *Comparative Studies in Society and History*, 52, 1 (2010), 180–209; Christian W. McMillen, *Discovering Tuberculosis. A Global History 1900 to the Present* (New Haven: Yale University Press, 2015), 91–109; Brimnes, *op. cit.* (note 44), 148–82.

48 Mahler referred only indirectly to Raman’s opposition, and he left India before Rajagopalachari’s opposition had gained momentum, but it is inconceivable that he was not well informed about both episodes of dissent. See McMillen and Brimnes, *op. cit.* (note 47), 195–6.

49 Government of India, *op. cit.* (note 41), Chapter 25, paras 36–42. The references to domiciliary treatment also illustrates techno-centric optimism. That domiciliary treatment with antibiotic drugs was effective against tuberculosis was not established until 1959. See Amrith, *op. cit.* (note 5), 150–6; Brimnes, *op. cit.* (note 44), 183–209.

50
*Report, op. cit.* (note 21), II, 339–41, 356.

51
*Report, op. cit.* (note 21), II, 18, 339–41, 349–51, 356.

52
*Report, op. cit.* (note 21), II, 389.

53
*Report, op. cit.* (note 21), II, 25. In vol. II, on p. 42 the semi-skilled worker was referred to as an ‘insufficiently trained’ solution for ‘the short term’. The Committee was more positive toward sanitary inspectors, which India required in large numbers to ‘participate effectively in the practical application of modern hygiene to rural health conditions’. See vol. I, 171–2.

54 Raja, *op. cit.* (note 38), 10–11.

55
*Report, op. cit.* (note 21) I, 19

56
*Report, op. cit.* (note 21), II, 455–7, quotes from pp. 455, 456. On the somewhat aborted attempt to incorporate indigenous traditions of medicine into the nationalist narrative in early twentieth-century India, see for instance Poonam Bala, ‘“Nationalizing” Medicine: The Changing Paradigm of Ayurveda in British India’ and Shamshad Khan ‘Colonial Medicine and Elite Nationalist Responses in India: Conformity and Contradictions’, both in Poonam Bala (ed.), *Contrasting Colonial Authority. Medicine and Indigenous Responses in Nineteenth and Twentieth-Century India* (Lanham: Lexington Books, 2012), 1–12 and 69–80.

57
*Report, op. cit.* (note 21), II, 353.

58
*Report, op. cit.* (note 21), I, 170–1; II, 396–402, quotes from pp. 397, 399.

59 Nehru, *op. cit.* (note 29), 543, 545.

60 Rajkumari Amrit Kaur, ‘Address to the Third Health Ministers Conference’, reproduced in *People’s Health*, IV, 12 (1950), 497–504, quoted from p. 498. I am grateful to the Adyar Library and Research Centre, Chennai for providing scans of this text.

61 Kaur, *ibid.*, 498–500.

62 Raja, *op. cit.* (note 38), 21–3. Quoted from p. 21 and 23.

63 Raja, *op. cit.* (note 38), 23.

64 Article from *The Hindu*, reproduced in *People’s Health*, IV, 12 (1950), 505–8.

65 Government of India, *op. cit.* (note 30), paras 84–88. Quoted from para 84.

66 Government of India, *op. cit.* (note 41), para 30.

67 In his final report from the BCG vaccination campaign, Mahler did refer to the Report of the Bhore Committee, along with references to the five-year plans. Halfdan Mahler, ‘Final Report on India BCG’, 3. Box CF/RA/BXPD/1962/T008, folder A124, United Nations Archive (UNICEF), New York.

68 My analysis is based on the views that Mahler expressed in his official correspondence. To the dismay of many public health historians, Mahler did not leave a private archive. Thanks to the kind cooperation of his son, Per Bo Mahler, I have been able to go through substantial parts of his private, unorganized papers, but I have not found much of relevance to the theme of this article. Mahler was known to write frank reports, in which he did not merely state what his superiors wanted to hear. UNICEF’s Regional Director noted, for instance, that Mahler’s final report contained remarks so ‘spicy that they are certain to be deleted out.’ S. Keeny (Bangkok) to N. R. Bowles (New York), 7. July 1955. Box CF/RA/BX/PD/1962/T008, folder A124, United Nations Archive (UNICEF), New York.

69 Halfdan Mahler, ‘Some of the conditions necessary for the development of an efficient mass vaccination programme’, Speech delivered at a BCG Conference in New Delhi, 18 December 1952, 7–8. Box CF/RA/BX/PD/1962/T008, folder A122, United Nations Archive (UNICEF), New York.

70 Halfdan Mahler, ‘India B.C.G. Project. Quarterly Field Report, 4^th^ Quarter 1953, 19 January 1954, 4. Box CF/RA/BX/PD/1962/T008, folder A122, United Nations Archive (UNICEF), New York.

71 Mahler, *op. cit.* (note 67), 20.

72 Halfdan Mahler, ‘India B.C.G. Project. Quarterly Field Report, 1^st^ Quarter 1954, 23 April 1954 5. Box CF/RA/BX/PD/1962/T071, folder B178; ‘India B.C.G. Project. Quarterly Field Report, 2^nd^ Quarter 1954’, 25 July 1954, 2. Box CF/RA/BX/PD/1962/T008, folder A122; ‘India B.C.G. Project. Quarterly Field Report, 4^th^ Quarter 1954’, 20. January 1955, 8. Box CF/RA/BX/PD/1962/T008, folder A122. All in United Nations Archive (UNICEF), New York.

73 Mahler, ‘1^st^ Quarter 1954’, *ibid.* 5; Mahler, ‘4^th^ Quarter 1954’, *ibid.*, 5.

74 Mahler, *op. cit.* (note 67), 21.

75 Engh, *op. cit.* (note 34).

76 Halfdan Mahler, ‘India B.C.G. Project. Progress Report for the Period from July 1952 to June 30, 1953’, 14-15. Box CF/RA/BX/PD/1962/T008, folder A122. For Mahler’s reference to ‘the present passive health-outlook of the villagers’, see Halfdan Mahler, ‘India B.C.G. Project. Quarterly Field Report, 3^rd^ Quarter 1953, 20 October 1953, 2. Box CF/RA/BX/PD/1962/T008, folder A122. See also Mahler, *op. cit.* (note 67), 9. All in United Nations Archive (UNICEF), New York.

77 Mahler, ‘2^nd^ Quarter 1954’, *op. cit.* (note 72), 10.

78 Mahler, *op. cit.* (note 67), 10, 15.

79 Mahler, ‘4^th^ Quarter 1954’, *op. cit.* (note 72), 4.

80 Mahler ‘2^nd^ Quarter,1954’ *op. cit.* (note 72), 9.

81 Mahler, *op. cit.* (note 67), 30, 39.

82 Mahler, *op. cit.* (note 67), 3. Emphasis in original.

83 Mahler, *op. cit.* (note 67), 8.

84 Mahler, *op. cit.* (note 67), 17. For the problematic evidence of the efficacy of BCG, see Brimnes, *op. cit.* (note 44), 81–83, 148–82, 259–63. See also Linda Bryder, ‘We shall not find Salvation in inoculation: BCG Vaccination in Scandinavia, Britain and the USA, 1921–1960, *Social Science & Medicine*, 49, 9. (1999),1157–67; Niels Brimnes, ‘BCG Vaccination and WHOs strategy for Tuberculosis Control 1948–83’, *Social Science & Medicine*, 67, 5 (2008), 963–73.

85 ‘Plan of operation for National Tuberculosis Programme, India’, June 1959, IND-MBD-003-1958-69, WHO Archives, Geneva. See also Sunil S. Amrith, ‘In Search of a “Magic Bullet” for Tuberculosis: South India and beyond’, *Social History of Medicine*, 17, 1 (2004), 113–30. Brimnes, *op. cit.* (note 44), 210–24.

86 ‘Technical Outlook and Programme of the National Tuberculosis Institute’, NTI, Bangalore, Technical Report Series No. 2, August 1959; ‘WHO Special Report: India’s National Tuberculosis Programme as a Problem of Social Planning’, n.d., both in IND-MBD-003, WHO Archives, Geneva. According to the cover letter, at least the first report was prepared by the international staff of NTI. The second report contained passages, which were repeated ad verbatim in an article written in Mahler’s name. See H. Mahler , ‘The WHO and the Ideas behind the Institute’, Bulletin Devoted to the Prevention of Tuberculosis, 7, 3 (1960), 7–12 .

87 ‘WHO Special Report’, *ibid.*, 3.

88
*ibid.*, 4, 8–9.

89
*ibid.*, 5–6.

90
*ibid.*, 5, 8, 35, 36.

91 This conclusion was based on unrealistically high expectations to both the coverage and efficacy of BCG vaccination. The efficacy of BCG had not been established by 1959 and the NTI reports urged that a major controlled trial of BCG be conducted. A trial was conducted a decade later with the surprising (and depressing) result that no protective effect of BCG against pulmonary tuberculosis in adults could be demonstrated under conditions prevailing in India. See Brimnes, *op. cit.* (note 44), 259–63; Brimnes *op. cit.* (note 84).

92 ‘Technical Outlook and Programme’, *op. cit.* (note 86), 1.

93 ‘WHO Special Report’, *op. cit.* (note 86), 30, 37.

94 ‘Technical Outlook and Programme’, *op. cit.* (note 86), 13.

95 ‘Technical Outlook and Programme’, *op. cit.* (note 86), 15.

96 ‘The National Tuberculosis ’Institute of India “Avalon”, Prospectus (1961)’, 1, IND MBD-003, WHO Archives.

97 See for instance NTI, research protocols RP22/1, 22/2, 23/1 and 23/2, IND MBD-003, WHO Archives.

98 WHO special Report’, *op. cit.* (note 86), 4, 32–3. For a key contribution to the sociology of tuberculosis control, see S. Andersen and D. Banerji, ‘A sociological Enquiry into an Urban Tuberculosis Control Programme in India, *Bulletin of the World Health Organization*, 29 (1963), 685–700.

99 Banerji, *The Making of A Community Health Physician in India: An intellectual Autobiography* (Delhi: Lok Prakash 2017), 3, 41.

100
*ibid.*, 74–5.

101 D. Banerji, ‘Medical Practice in India and Its Sociological Implications’, *The Antiseptic* (February 1962), 125–9, quoted from 126–8. Banerji would later laud the Alma-Ata Declaration as a ‘watershed’ in the history of public health and a triumph of the masses around the world. See Niels Brimnes, ‘Fallacy, sacrilege, betrayal and conspiracy: the cultural construction of opposition to immunisation in India’, in C. Holmberg, S. Blume, and P. Greenough, *The politics of vaccination. A global history* (Manchester: Manchester University Press, 2017), 51–76. See particularly pp. 65–9 and Banerji, *op. cit.* (note 99), 192.

102 Litsios, *op. cit.* (note 6), 716.

103 Interview with Daniel Tarantola, by Marcos Cueto, Theodore M. Brown and Niels Brimnes, conducted online 15. June 2021.

